# Supplementary material for: Self-supervised learning for label-free segmentation in cardiac ultrasound
Source: Nat Commun. 2025 Apr 30;16:4070. doi: 10.1038/s41467-025-59451-5 (PMC12043926; doi:10.1038/s41467-025-59451-5)
Supplement: Supplementary file 1 — Supplementary Information [file 41467_2025_59451_MOESM1_ESM.pdf]

Title: Self-supervised learning for label-free segmentation in cardiac ultrasound

Authors: Danielle L. Ferreira PhD, Connor Lau, Zaynaf Salaymang RDCS, Rima Arnaout MD

Supplemental Methods

Data preprocessing and initial weak label extraction. *Initial weak label extraction, A2C view.* A bilateral filter ($\sigma_s = 15$, $\sigma_r = 0.25$) was applied to images for speckle noise reduction[1]. A Euclidean distance transform (minimum distance = 20 pixels) was then applied to propose seed points for the watershed algorithm. The watershed algorithm[2] was applied to create initial segments. A copy of this image was binarized (threshold = 0.1) to create a mask for the blood pool, and this mask was applied to remove watershed segments outside the blood pool. Next, connected components analysis excluded images for which the watershed algorithm failed to find at least two segments (since two chambers are expected in the A2C view). The remaining suitable segments were labeled as 'LA' or 'LV' according to the known spatial relationship of these chambers in the A2C view (using clinical knowledge of the A2C view, we expect to see LA in the bottom of the image and the LV above it) . Finally, shape descriptors such as eccentricity and area (see "quality control" below for thresholds used) were calculated, compared with clinical knowledge about plausible chamber shapes and sizes, and were used to eliminate segments not compatible with known anatomy.

Initial weak label extraction, A4C view. The final UNet trained for A2C prediction was used to predict segmentations for A4C images (resulting in predictions with two LAs and two LVs per image). Centroids were calculated for each segment and clinical knowledge about known anatomical relationships leveraged to re-label these chambers as RA, RV, LA, and LV. Shape and topological descriptors derived from aggregate clinical knowledge were used to discard images whose segmentations failed relationship, size, and eccentricity thresholds (see "quality control" below), and which lacked four connected components with the expected geometric relationships of the four chambers.

During pipeline training for A4C, intermediate predictions on the validation set were noted to have systematically shorter RV length than appropriate given clinical knowledge about LV-RV length relationships. We suspected this was due to intermediate predictions' failure to correctly understand RV trabeculations. Intermediate predictions were therefore refined based on clinical

spatial priors as follows. Length of the predicted LV and RV segments were measured, and its ratio β (RV height/LV height) computed. If the ratio β was less than 0.8, that is RV height was 80% of the LV height value or smaller, the RV segment was stretched to be proportional to the LV.

Initial weak label extraction, SAX view. The Hough circle transform[3] was used to leverage clinical knowledge that the LV in the SAX view has a circular shape. A median blur filter (kernel size = 9) was applied to images to reduce noise and a Laplacian operator (kernel size = 5) was employed to extract edges and to reduce the amount of data in the image, an important step to reduce the computation time of the Hough transform. Finally, the Hough circle transform[3] was applied to detect the center and the radius of the endocardial region. Parameters for the Hough transform were a minimum distance between the centers of the detected circles of 400, minimum and maximum circle radius of 20 and 80, respectively; these were chosen empirically based on the approximate size of the LV as a proportion of the image in the SAX view.

For all hyperparameters used in data preprocessing and initial weak label extraction, hyperparameters across a range of 0.1X to 10X of default values were tested on a small sample (n~20) of images. The best parameters chosen by visual review and quantification of what proportion of images had plausible weak labels generated under the various conditions.

Neural network architectures. *Quality control for segmentations for initial weak label extraction and during network training.* These quality control thresholds were derived from clinical knowledge about chamber shapes, sizes, and relationships. A2C: Cutoffs for area were 6 to 75cm^2 for LA and RA, and 4.7 to 104cm^2 for LV and RV. Acceptable eccentricities were 0.3-0.96 for LA, 0.17-0.95 for RA, 0.62-0.96 for LV, and 0.65-0.96 for RV. Connected components analysis required two chambers for the A2C view (an LA and an LV), and four chambers for the A4C view (LA, RA, LV, RV).

Table S1: Correlation, bias, accuracy, and kappa values.

Test set	Comparison	N	Regression		BA analysis		ICC	Normal vs Abnormal			
**LVEDV (mL)**			**r**	**r^2**	**Bias**	**LOA**		**Acc**	**Kappa**	**F1**	**AUC**
All-comers	Echo vs AI	5648	0.84	0.70	2.77mL	±50.9mL	0.83	0.87	0.57	0.65	0.80
CMR subset	Echo vs SSL	468	0.83	0.69	2.48mL	±63.5mL	0.81	0.8	0.57	0.72	0.80
CMR subset	CMR vs SSL	468	0.78	0.60	-57.64mL	±108.6mL	0.41	0.78	0.52	0.69	0.77
CMR subset	CMR vs Echo	468	0.82	0.67	-60.13mL	±95.6mL	0.50	0.83	0.61	0.74	0.81
Clinical variability	literature	50	--	--	23.0mL	±42mL	--	--	--	--	--
Supervised learning	literature	337	--	0.7	2.3mL	±58mL	--	--	--	--	--
Supervised learning	literature	8457	--	0.65	2.0mL	±56mL	--	--	--	--	--
**LVESV (mL)**											
All-comers	Echo vs SSL	5648	0.9	0.82	5.3mL	±31.6mL	0.88	0.81	0.54	0.66	0.83
CMR subset	Echo vs SSL	468	0.9	0.81	4.24mL	±41.25mL	0.88	0.81	0.62	0.79	0.83
CMR subset	CMR vs SSL	468	0.85	0.73	-30.06mL	±94.6mL	0.60	0.79	0.57	0.79	0.79
CMR subset	CMR vs Echo	468	0.88	0.77	-34.29mL	±83.3mL	0.66	0.78	0.57	0.76	0.79
Clinical variability	literature	50	--	--	11mL	±20mL	--	--	--	--	--
Supervised learning	literature	337	--	0.74	3.6mL	±41mL	--	--	--	--	--
Supervised learning	literature	8457	--	0.74	--	±39mL	--	--	--	--	--
**LVEF (%)**											
All-comers	Echo vs SSL	5648	0.81	0.65	-5.27%	±14.6%	0.72	0.97	0.79	0.81	0.93
CMR subset	Echo vs SSL	468	0.9	0.8	-3.97%	±14.1%	0.86	0.95	0.83	0.86	0.94
CMR subset	CMR vs SSL	468	0.8	0.63	-1.33%	±21.8%	0.78	0.91	0.68	0.74	0.82
CMR subset	CMR vs Echo	468	0.83	0.68	2.63%	±20.3%	0.81	0.91	0.69	0.74	0.81
Clinical variability	literature	50	--	--	8%	±18%	--	--	--	--	--
Supervised learning	literature	337	--	0.5	-1.9%	±21%	--	--	--	--	--
Supervised learning	literature	8457	--	0.46	-5%	±20%	--	--	--	--	--
**LV mass (g)**											
All-comers	Echo vs SSL	5128	0.74	0.55	13.7g	±73.6g	0.71	0.71	0.35	0.56	0.70
CMR subset	Echo vs SSL	42	0.84	0.71	13.3g	±72.6g	0.83	0.86	0.71	0.86	0.86
CMR subset	CMR vs SSL	42	0.65	0.42	35.3g	±122g	0.55	0.61	0.25	0.6	0.63
CMR subset	CMR vs Echo	42	0.59	0.35	21.7g	±130g	0.56	0.62	0.24	0.58	0.62
Clinical variability	literature	60	--	--	8.7g	±49g	--	--	--	--	--
Supervised learning	literature	337	--	--	--	±91g	--	--	--	--	--
**LA volume (mL)**											
All-comers	Echo vs SSL	2168	0.92	0.84	-0.15mL	±20.1mL	0.92	0.9	0.77	0.83	0.88
Clinical variability	literature	85	--	--	7mL	±13mL	--	--	--	--	--
Supervised learning	literature	4800	--	--	5mL	±3mL	--	--	--	--	--
**RVEDA (cm^2)**											
All-comers	Echo vs SSL	381	0.83	0.69	-0.86cm^2	±5.4cm^2	0.80	0.91	0.54	0.59	0.79
**RVESA (cm^2)**											
All-comers	Echo vs SSL	381	0.84	0.71	1.60cm^2	±3.9cm^2	0.75	0.72	0.26	0.36	0.85
**RA volume (mL)**											
All-comers	Echo vs SSL	1007	0.87	0.76	-2.10mL	±21mL	0.87	0.92	0.73	0.78	0.86

Echo = clinical echocardiogram measurements, SSL = self-supervised AI pipeline-derived measurements, LVEF = left ventricular ejection fraction, LVEDV = left ventricular end-diastolic volume, LVESV = left ventricle end-systolic volume, RVEDA = right ventricular end-diastolic area, RVESA = right ventricular end-systolic area, r = Spearman correlation coefficient, BA = Bland-Altman, LOA = 95% limits of agreement (two standard deviations), ICC = intra-class correlation, AUC = area under the receiver-operator curve. CMR = cardiac MRI. As in main Figure 3, clinical intra-observer references include Jacobs et al 2006 for LVEDV, LVESV, and LVEF[14]; Crowley et al 2016 for LV mass[52]; and Mihaila et al 2017 for LA volume. Supervised learning references include Ghorbani et al 2020[41] and Zhang et al 2018[7].

Table S2. Model performance of LV segmentation in an external dataset.

Dataset	EchoNet dataset (Nb patients)	Average Dice Score, Diastole [95% CI*]	Average Dice Score, Systole [95% CI*]
EchoNet model†	EchoNet test set (1,277)	0.93 [0.93 - 0.93]	0.90 [0.90 - 0.91]
Our model	EchoNet test set (1,277)	0.89 [0.89 - 0.89]	0.85 [0.85 - 0.85]
	Whole dataset (10,030)	0.89 [0.89 - 0.89]	0.85 [0.85 - 0.85]

Nb = number
* Confidence intervals were computed using 10,000 bootstrapped samples
† Values for EchoNet test set were extracted directly from the paper[8] since the EchoNet model is not available.

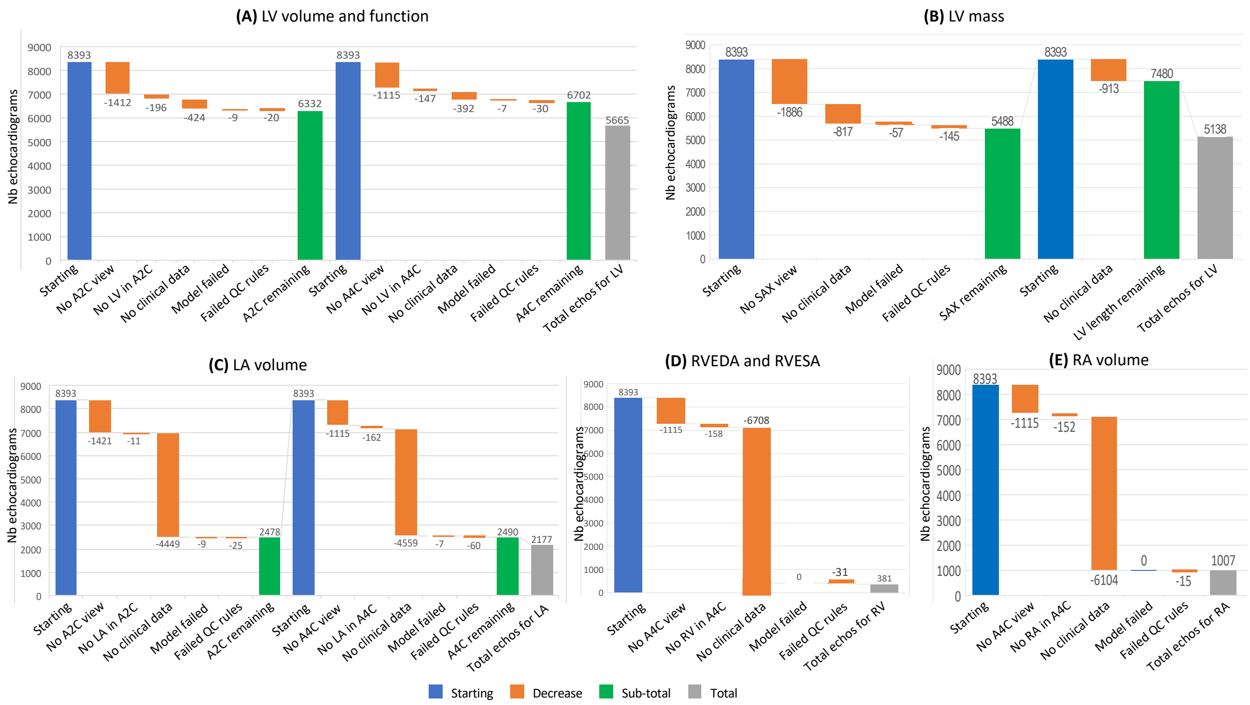

Figure S1. Inclusion/exclusion of test data by clinical measurement. The test set had 8,393 echocardiograms/patients. For each measurement assessed, certain views and chambers needed to be present in the echocardiogram as above; a clinical measurement needed to be present in the echocardiogram report for ground-truth comparison. (A) shows the echocardiograms containing A2C and A4C views and clinical ground-truth measurements of the LV for LV size and function assessments. (B) shows the echocardiograms containing SAX views of the LV and LV length measurement for LV mass assessment. (C) shows the echocardiograms containing A2C and A4C views/measurements of the LA, while (D) and (E) show the A4C views/measurements needed for RV and RA assessment, respectively. LV = left ventricle, LA = left atrium, RV = right ventricle, RA = right atrium, QC = quality control, A2C = apical 2-chamber view, A4C = apical 4-chamber view, SAX = short-axis mid view, Nb = number. “No clinical data” = no clinical echocardiogram measurement for comparison to ground truth, “Model failed” = model did not predict anything, “Failed QC rules” = model predicted a chamber, but its shape and/or size failed post-processing rules.

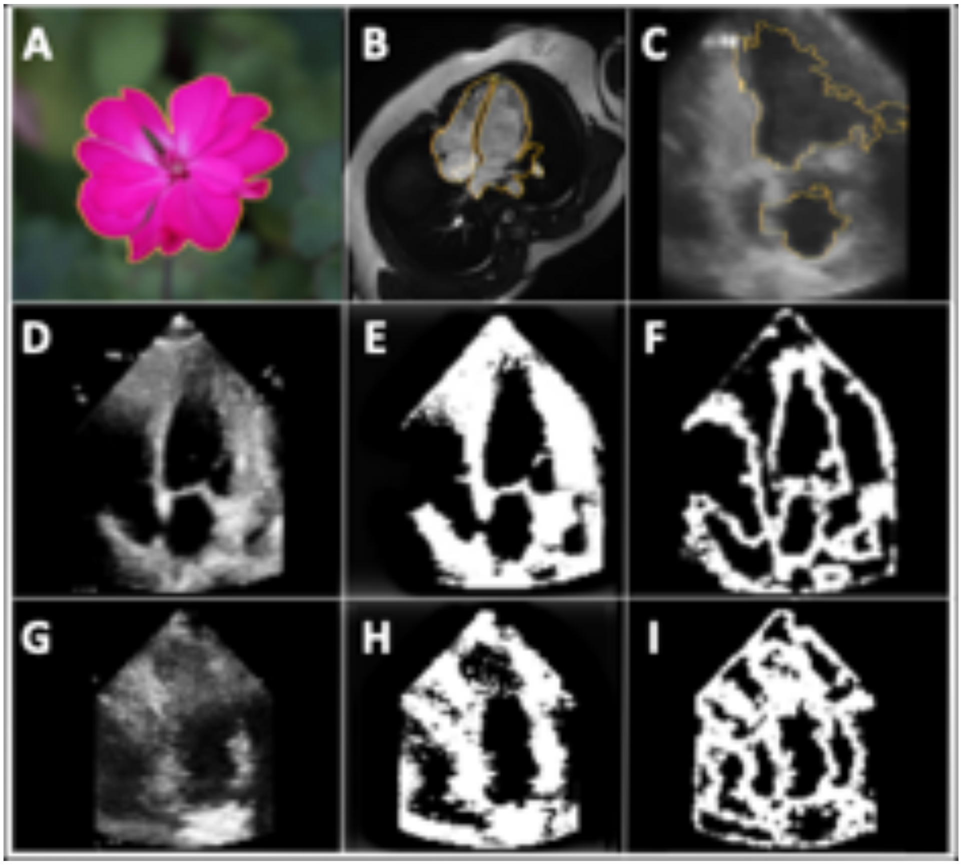

Figure S2. Examples of standard computer vision methods on different images. First row – classical computer vision watershed algorithm (thin yellow line) applied to (A) a photo image of a flower, and medical images of the heart in (B) MRI and (C) ultrasound modalities. Note that this algorithm works best on a photo image. Second and third rows depict two echo image examples – left (D,G): original echo images, middle (E,H): segmentation using bilateral filtering, right (F,I): segmentation using optical flow between consecutive frames of the echocardiography sequence.

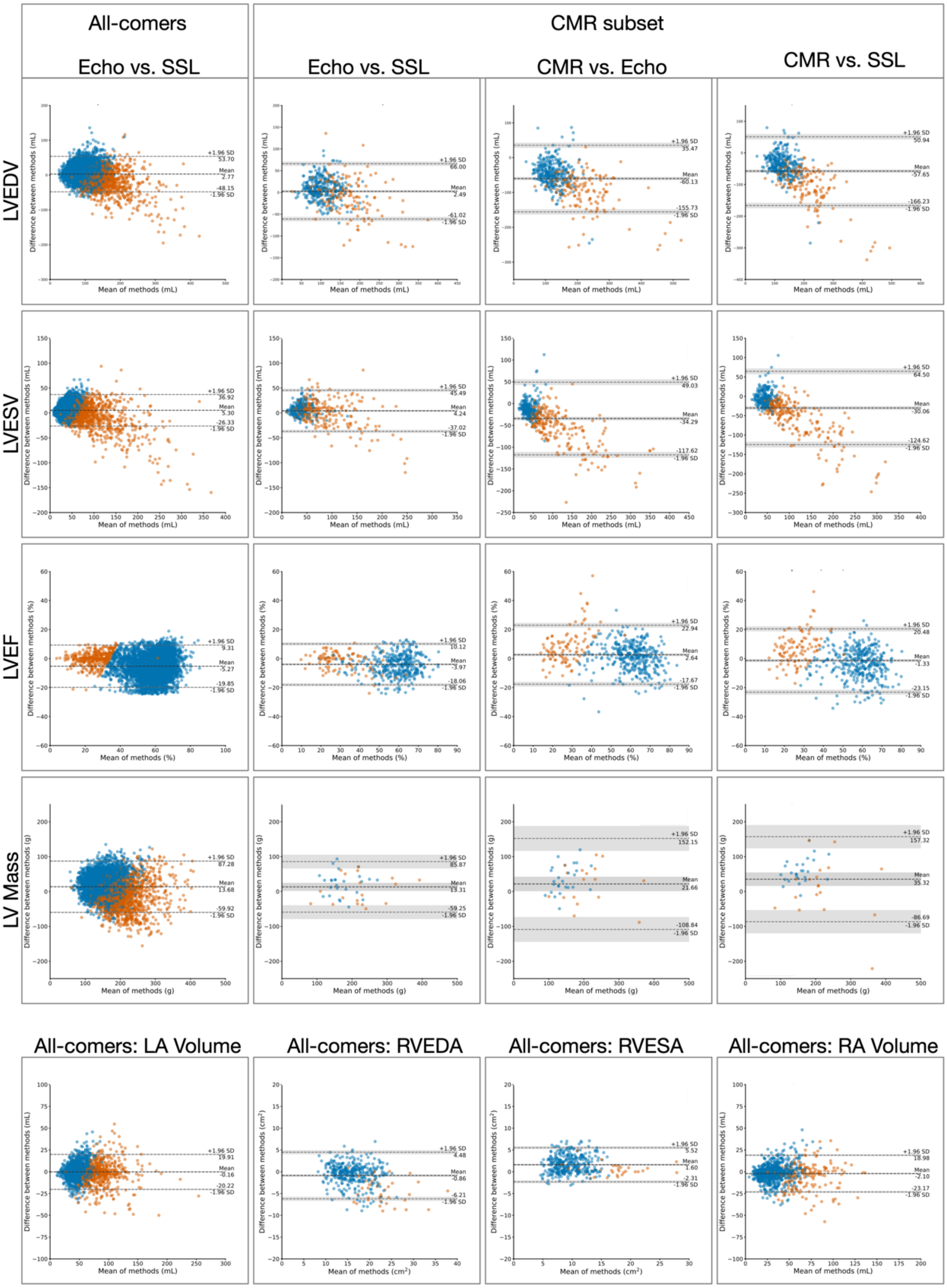

Fig S3: Bland-Altman plots. Blue dots represent normal measurements; orange dots, abnormal. SSL = self-supervised AI pipeline, CMR = cardiac MRI, LVEDV= LV diastolic volume, LVESV = LV systolic volume, LVEF = LV ejection fraction, RVEDA and RVESA = RV end-diastolic and -systolic areas, respectively. Source data are provided as a Source Data file.

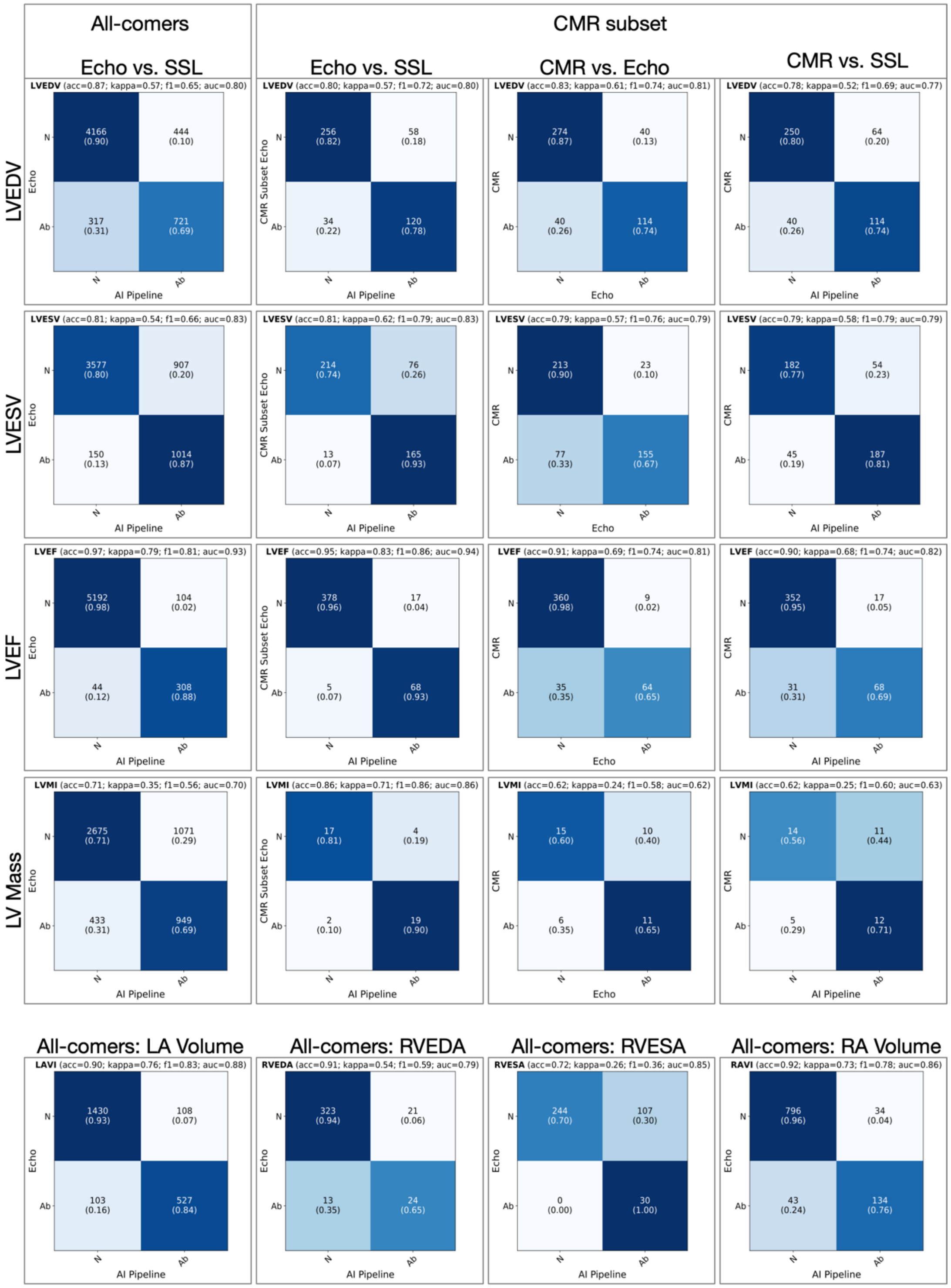

Fig S4: Confusion matrices for measurements binarized into normal (N) vs. abnormal (Ab). Accuracy and Cohen's kappa are shown. Measurements are indexed by body surface area where clinically applicable. SSL = self-supervised AI pipeline, CMR = cardiac MRI, LVEDVI = LV diastolic volume index, LVESVI = LV systolic volume index, LVEF = LV ejection fraction, LVMI = LV mass index, RVEDA and RVESA = RV end-diastolic and -systolic areas, respectively, RAVI = RA volume index. Source data are provided as a Source Data file.

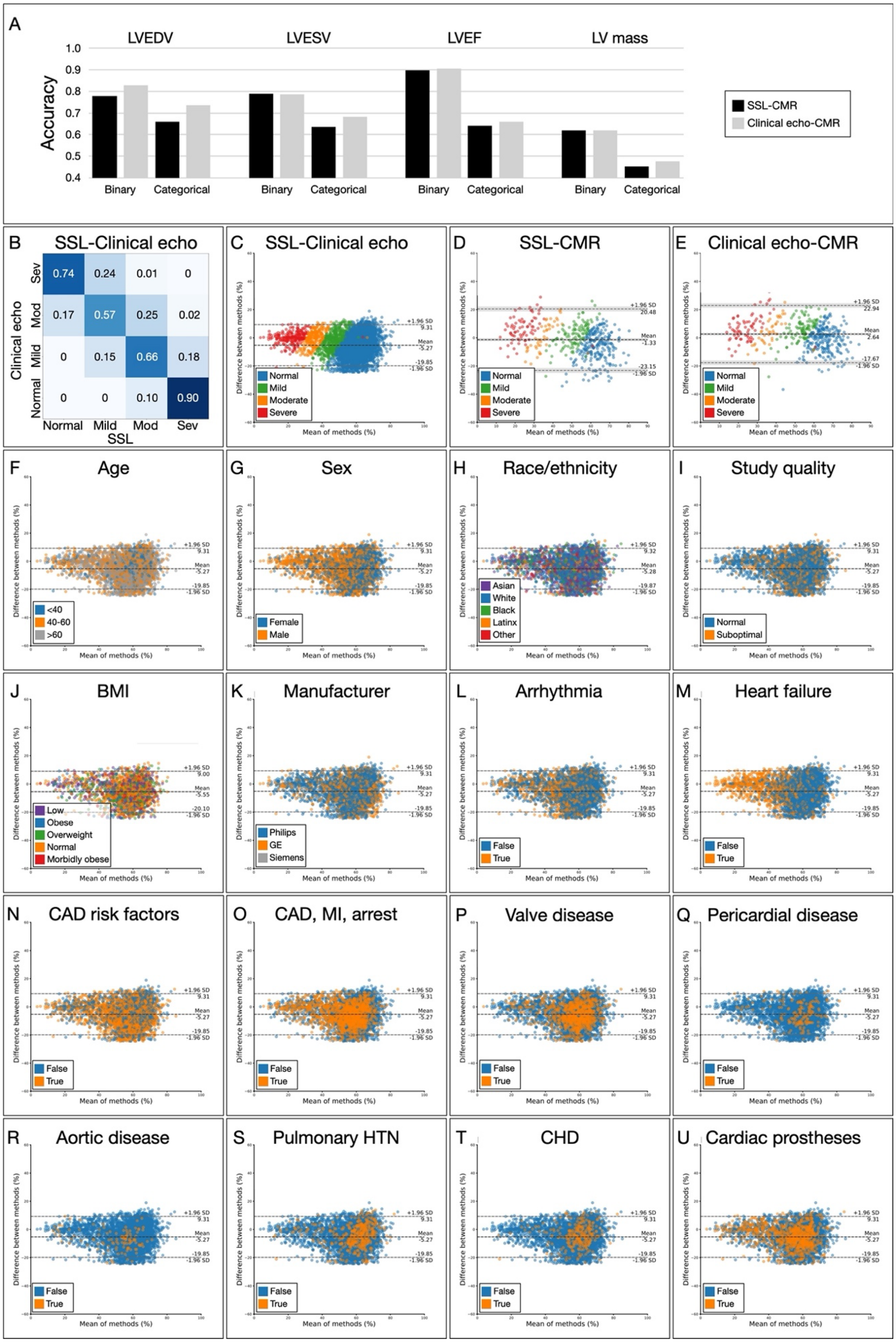
A
LVEDV
LVESV
LVEF
LV mass
Accuracy
1.0
0.9
0.8
0.7
0.6
0.5
0.4
Binary
Categorical
SSL-CMR
Clinical echo-CMR
B
SSL-Clinical echo
Clinical echo
Sev
Mod
Mild
Normal
0.74 0.24 0.01 0
0.17 0.57 0.25 0.02
0 0.15 0.66 0.18
0 0 0.10 0.90
Normal Mild Mod Sev
SSL
C
SSL-Clinical echo
D
SSL-CMR
E
Clinical echo-CMR
Normal
Mild
Moderate
Severe
F
Age
<40
40-60
>60
G
Sex
Female
Male
H
Race/ethnicity
Asian
White
Black
Latinx
Other
I
Study quality
Normal
Suboptimal
J
BMI
Low
Obese
Overweight
Normal
Morbidly obese
K
Manufacturer
Philips
GE
Siemens
L
Arrhythmia
M
Heart failure
N
CAD risk factors
O
CAD, MI, arrest
P
Valve disease
Q
Pericardial disease
R
Aortic disease
S
Pulmonary HTN
T
CHD
U
Cardiac prostheses
False
True
Difference between methods (%)
Mean of methods (%)

Figure S5. Performance by categorical values, patient factors, and cardiac pathology. (A). Accuracy of SSL-derived (black) and clinical (grey) measurements compared to CMR gold standard according to binary breakdown of normal vs abnormal or categorical breakdown by normal, mild, moderate, severe. Accuracies for binary classifications were higher than categorical accuracies for both clinical and SSL-derived measurements (p=0.008). There was no statistically significant difference between clinical and SSL-derived accuracies (p=0.66). Confusion matrix (B) and Bland-Altman plots (C-E) showing categorical performance for LV ejection fraction (LVEF). (C) shows SSL performance compared to clinical echocardiography measurements. (D) and (E) compare SSL and clinical measurements to the CMR gold standard. (F-U) Bland-Altman plots of SSL performance against clinical measurements, colored by patient demographics, study quality, and cardiac pathologies as indicated. LVEDV, left ventricular end-diastolic volume. LVESV, left ventricular end-systolic volume. SSL, self-supervised learning. CMR, cardiac MRI. CAD, coronary artery disease. MI, myocardial infarction. HTN, hypertension. CHD, congenital heart disease. Cardiac prostheses includes pacemakers, grafts, balloon pumps, ventricular assist devices. Source data are provided as a Source Data file.

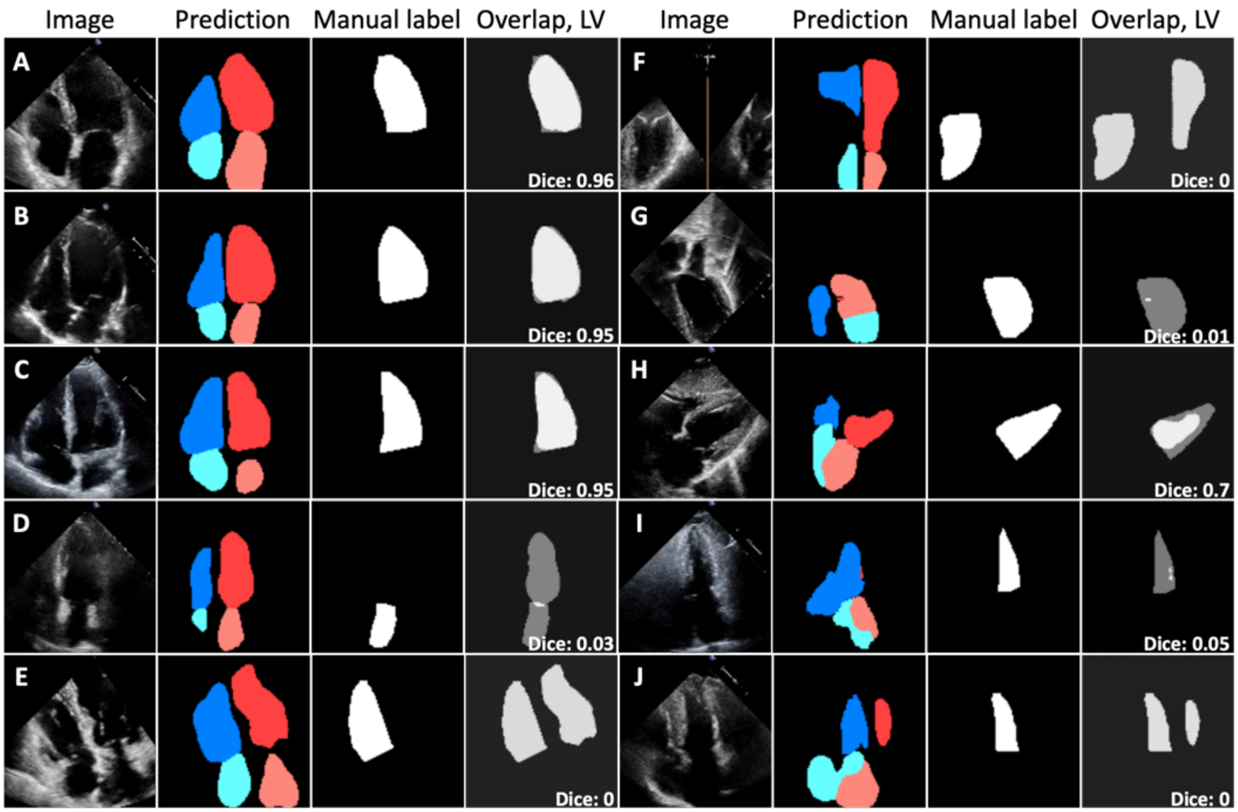

Figure S6. AI pipeline performance on A4C images from an external dataset: best- and worst-case examples. This dataset had only A4C images and manually annotated clinical labels for only the left ventricle available. Dice scores ("Overlap, left ventricle") between the AI pipeline's LV segmentation ("AI pipeline prediction," red) and the manual LV segmentation ("EchoNet manual label") are shown. Average Dice score between AI pipeline and manual labels was 0.89 over twenty thousand images. This average Dice includes examples where AI pipeline matched manual label well with high per-image Dice scores (A-C); examples where the AI pipeline was correct but the manual label incorrectly labeled the left atrium (D) or the right ventricle (E) leading to extremely low per-image Dice scores. In these cases, the model was in fact correct despite the low Dice scores. Overall performance also included image view types in the external dataset that that were not part of the AI pipeline training, such as split view (F), inverted view (G), subcostal view (H), and A2C view classified as A4C (I). As these were cases of mislabled views, good model performance was not expected. Finally, it included rare examples where the view and the manual label were correct and the AI pipeline failed (J).

Supplementary References

1. Wu S, Zhu Q, Xie Y. Evaluation of various speckle reduction filters on medical ultrasound images. Annu Int Conf IEEE Eng Med Biol Soc 2013;2013:1148–1151. doi:10.1109/EMBC.2013.6609709.
2. Roerdink JBTM, Meijster A. The Watershed Transform: Definitions, Algorithms and Parallelization Strategies. Fundamenta Informaticae 2000;41:187–228.
3. Yuen H, Princen J, Illingworth J, Kittler J. Comparative study of Hough Transform methods for circle finding. Image and Vision Computing 1990;8:71–77. doi:10.1016/0262-8856(90)90059-E.
